# Mapping and size estimation of men who have sex with men in virtual platforms in Delhi, India

**DOI:** 10.1371/journal.pone.0262094

**Published:** 2022-01-20

**Authors:** Shajy Isac, Purnima Parmar, Carl Boodman, Shishram Ola, Reynold Washington, J. K. Mishra, Parveen Kumar, Marissa Becker

**Affiliations:** 1 India Health Action Trust, New Delhi, India; 2 Institute for Global Public Health, Max Rady College of Medicine, University of Manitoba, Winnipeg, Manitoba, Canada; 3 Department of Community Health Sciences, Max Rady College of Medicine, University of Manitoba, Winnipeg, Manitoba, Canada; 4 National AIDS Control Organisation, Ministry of Health & Family Welfare, New Delhi, India; 5 Department of Internal Medicine, Section of Infectious Diseases, Max Rady College of Medicine, University of Manitoba, Winnipeg, Manitoba, Canada; 6 Department of Medical Microbiology and Infectious Diseases, Max Rady College of Medicine, University of Manitoba, Winnipeg, Manitoba, Canada; 7 Delhi State AIDS Control Society, New Delhi, India; UNSW Australia, AUSTRALIA

## Abstract

**Introduction:**

In India, the HIV epidemic is concentrated among Key Populations (KPs), such as men who have sex with men (MSM), who bear a disproportionate burden of HIV disease. Conventional targeted interventions (TI) mitigate HIV transmission among MSM by focusing on physical hotspots. As increasingly, there is a shift within India’s MSM community to connect with sex partners online, novel approaches are needed to map virtual platforms where sexual networks are formed. The objective of this study was to estimate the number of MSM in Delhi using virtual platforms to connect for sex and to describe patterns of their use.

**Methods:**

The study was conducted in the state of Delhi among MSM over 18 years of age who used virtual platforms to look for sexual partners. Virtual platforms were identified through community consultations. Size estimation was carried out by enumerating the number of online users, accounting for duplication across sites and time and based on interviews with 565 MSM.

**Results:**

28,058 MSM (95% CI: range 26,455–29,817) use virtual sites to find sexual partners. We listed 14 MSM specific virtual sites, 14 general virtual sites, 19 social networking pages and 112 messenger groups, all used by MSM. Five virtual sites met feasibility criteria to be included in the virtual mapping. Of the MSM on these sites, 81% used them at night and 94% used them on Sundays, making these the peak time and day of use. Only 16% of users were aware of organizations providing HIV services and 7% were contacted by peer educators in the preceding three months. Two-fifths (42%) also visited a physical location to connect with sexual partners in the month prior to the study.

**Discussion:**

TI programs that focus on physical hotspots do not reach the majority of MSM who use virtual sites. MSM active on virtual sites have a low awareness of HIV services. Virtual mapping and programmatic interventions to include them must be incorporated into current public health interventions to reach MSM at risk of HIV.

## Introduction

Key populations (KP) account for a disproportionate burden of human immunodeficiency virus (HIV) infections globally [1]. In Asia and the Pacific, KP account for 98% of the 300,000 new HIV infections [1]. Men who have sex with men (MSM) are 26 times more likely to acquire HIV than the general population [1]. The elevated HIV rate among MSM is multifactorial in origin: high-risk behaviour is perpetuated by homophobia, stigma and systemic discrimination that limits engagement with traditional HIV education, treatment and prevention programs [2,3]. This signals the need to develop innovative strategies that engage the MSM population with HIV prevention and treatment services.

In India, there are approximately 2.1 million people living with HIV, accounting for one of the biggest populations of people living with HIV in the world [1,4,5]. HIV prevalence among MSM is 2.69%, much higher than the prevalence of 0.28% within the general population [4,6]. Indian MSM remain extremely stigmatized, facing discrimination linked to their sexual orientation [7–9]. Many MSM in India marry women and have unprotected sex with their wives, thus acting simultaneously as a KP and a bridge population [7,10]. Despite the elevated risk of acquiring HIV, 47% of MSM in India have never been tested for HIV [11].

MSM increasingly utilize virtual platforms such as geosocial networking mobile phone applications to connect with sexual partners [12–14]. The more popular “apps” boast millions of users and over half of MSM in certain areas use virtual platforms to meet partners [15]. MSM often use virtual platforms in addition to connecting with partners in physical locations (“hotspots” such as street corners, brothels, hotels, etc.,) [16,17]. The use of virtual platforms is especially common among MSM living in countries with greater societal homophobia, as online platforms offer anonymity, control and a perception of safety [18]. While online connection for sex may protect MSM from discrimination, the use of virtual platforms is associated with other risks: MSM who seek sexual partners online report to be more likely to have unprotected sex, have more sexual partners and have a higher prevalence of sexually transmitted infections [12,15]. In India, the HIV risk of connecting online for sex is amplified by low rates of HIV status disclosure to partners met online, further emphasising the need for public health interventions that specifically cater to the online MSM community [19].

Targeted interventions (TI) are public health interventions that provide focused HIV prevention services to prioritized KP groups [20]. TIs provide high-risk populations with the information, means, skills and services they need for HIV prevention and care [20,21]. In Delhi, the National AIDS Control Organization (NACO) and the Delhi State AIDS Control Society (DSACS) have been implementing a TI program exclusively for MSM since 2008 [21]. Currently, NACO’s 11 MSM TI programs, operating throughout the eleven districts of Delhi, have provided HIV prevention and treatment services to 16052 MSM [22]. However, traditional TIs concentrate HIV prevention services at physical hotspots, but do not reach those who operate exclusively on virtual platforms that may act as online loci of HIV transmission [23–25]. While establishing the size of KPs is critical to provide services to an unreached population and to evaluate the impact of HIV intervention programs, conventional population size estimation at physical hotspots excludes MSM operating exclusively on virtual platforms and thus underestimates KP size [24–26].

The World Health Organization and the United Nations Program on HIV/AIDS recommend the inclusion of virtual mapping in the estimation of KPs [27]. However, this dimension of HIV programming remains largely unexplored in India. The Delhi State AIDS Control Society (DSACS), in collaboration with the Technical Support Unit-Delhi (DL TSU), which is implemented by India Health Action Trust (IHAT) and in partnership with the University of Manitoba, Canada, implemented a mapping exercise to determine the population size of MSM active on virtual platforms in Delhi. Here, we describe this virtual mapping approach as a method to estimate the population size of MSM in Delhi using virtual platforms and to generate an understanding of the level of HIV exposure associated with virtual platform use.

## Methods

### Study setting and study population

This study was conducted by the team of the DL-TSU, supported by DSACS and IHAT, in partnership with the University of Manitoba, Canada. The virtual mapping exercise was undertaken in Delhi, National Capital Territory (NCT). Eligible study participants were MSM living in Delhi, 18 years or older, who provided informed verbal or written consent to be interviewed during Stage 3. This population included, but was not limited to, MSM who acknowledged having exchanged sex in return for money, drugs or materials.

### Study design

**Fig 1:**
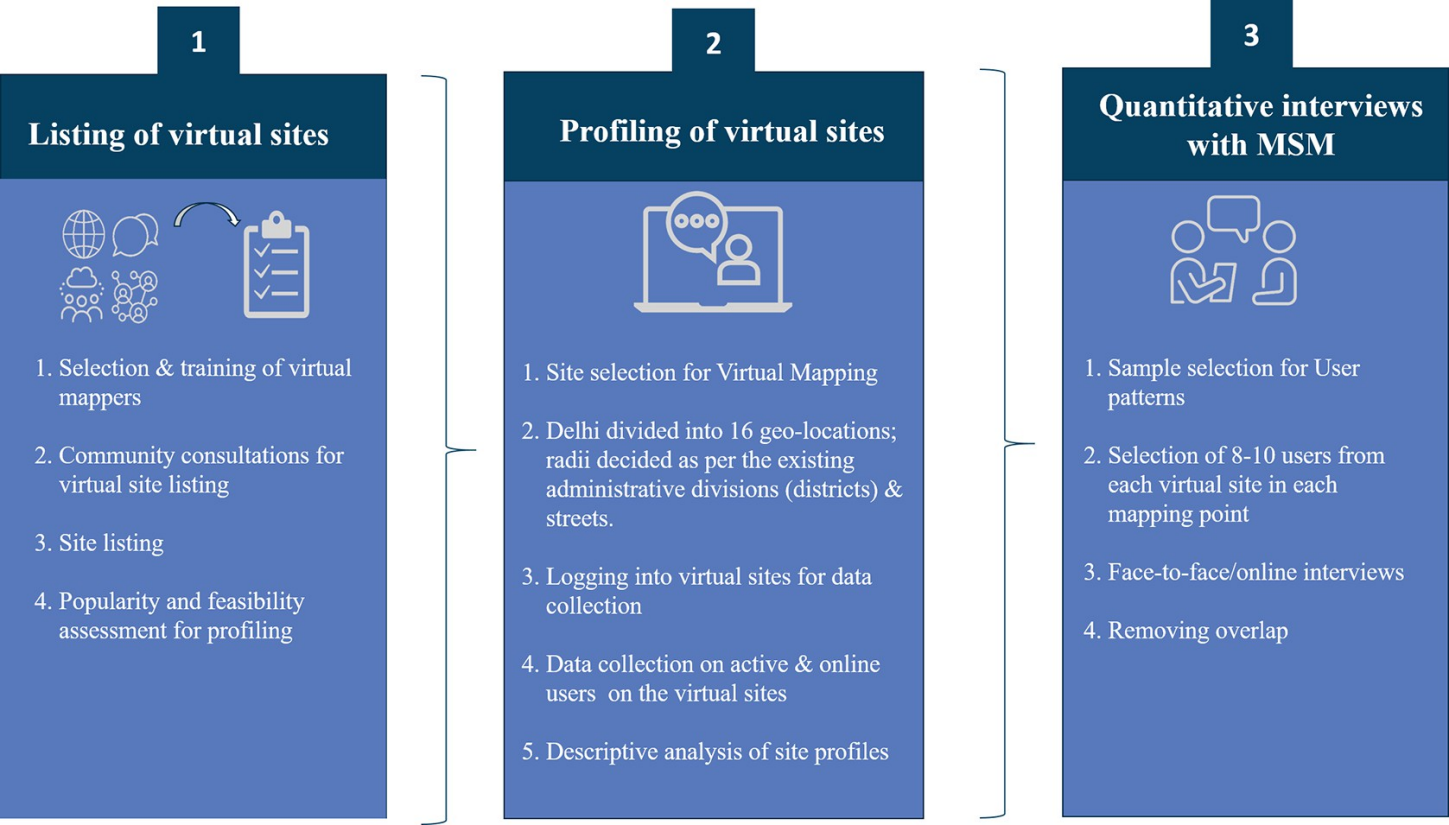
Stages of virtual mapping.

Virtual mapping was carried out in three stages (Fig 1). Stage 1 involved listing the virtual platforms used by MSM to connect with sexual partners. Stage 2 consisted of profiling and estimating the population size of MSM on each virtual platform. Stage 3 involved structured interviews to characterize MSM active on virtual platforms, including information on their use of virtual sites as well as use of physical hotspots to meet sexual partners, and to understand access to HIV prevention and treatment services.

### Data collection

#### Stage 1: Listing of virtual platforms

The DL TSU trained and mentored a team of four community members who self-identified as MSM, and who had extensive knowledge of virtual platforms, as Virtual Mappers (VMs). VMs undertook a two-day training on mapping processes, tools, interview techniques, ethics and data collection. These VMs conducted intensive consultations with 112 community members, who self-identified as MSM and who were active on virtual platforms, to gather information on names, types and features of the virtual platforms. A pre-structured format was used to collect the information, which included name and type of virtual platform, features such as being global positioning system (GPS)-enabled, websites being local or global and requirement of payment for registration, among other details. A comprehensive list of the various virtual platforms used by MSM was developed.

The listing revealed 14 MSM-specific and 14 general virtual sites (sites with mobile app or other internet pages), 19 social networking pages exclusive for MSM (eg. Facebook, Instagram etc) and 112 messenger groups (WhatsApp groups). The team (VMs, TI staff and community members) logged into popular platforms to determine the feasibility of their use in the mapping process.

A total of 30 virtual platforms were considered for the feasibility assessment, out of which 27 were virtual sites with either mobile applications or other websites, two were social networking pages and one was a messenger group. The feasibility for mapping was then determined based on the following criteria: (i) MSM-specific sites, (ii) availability of information on active and online users, and, (iii) an identifiable geo-location. Further, popularity of a platform was defined as the most commonly mentioned sites as per the consulting community members. Five virtual sites were found to be popular as well as feasible for mapping.

#### Stage 2: Profiling of virtual platforms

The second stage of mapping involved profiling the selected virtual platforms to estimate the MSM population size. Being GPS-enabled, these apps provided information on the number of active users and users online in a defined geographical location.

With the help of a map developer tool, namely Google Map developer, the map of Delhi NCT was divided into 16 geo-locations taking into account the administrative division (districts/wards) and streets in such a way that the entire geography was divided without overlap. Estimation of the size of the MSM population was done through manual counting of the active users on the virtual sites within a geo-location. In each geo-location, VM logged on to collect information during specific time periods through the day: morning (before 12 noon), afternoon (12 noon to 5 pm), evening (5 pm to 8 pm) and night (after 8 pm). In order to profile the platforms, VMs collected information on: name of the site, day and time of visit, total number of registered users, number of users online during the visit time, and location of the site or users.

#### Stage 3: Quantitative interviews with MSM

Determining whether individuals were active on multiple sites as well as determining multiple profiles of an individual on the same site was important to avoid duplication in size estimation. We conducted interviews with a representative sample of MSM in virtual sites to understand the profiles. Assuming a population prevalence of 50% with a precision of 5% and 95% confidence interval, the required sample size was 384. In each of the geo-locations, we included a sample of 8–10 participants per site (total 560 participants). Assuming a 15% non response, the sample was increased to 640. The list of virtual sites from stage 1 and the number of active MSM derived during Stage 2 formed the sampling frame. In each of the five virtual applications selected, and in each geo-location, a random sample of 8–10 MSM were selected for interviewing. Thus, 128 MSM from each of the five virtual sites identified were randomly selected (a total of 640 individuals). Of these individuals, 565 consented to participate. Individuals who provided consent were explained the purpose and objectives of the mapping exercise and process and agreed on a mutual time and place (in case of face-to-face interviews) for the interview. Non-response and incomplete interviews were captured in the log sheet. Virtual mappers collected information on the usage of virtual sites for meeting sexual partners, presence on multiple virtual sites, visit to physical hotpots, sexual identity, timing and day of operation in virtual sites and exposure to the TI program from each participated respondents. Sexual identity was captured as self-reported and included the following categories: “Panthi” (those who prefer an insertive role during sex), “double decker” (those who both insert and receive during sex), “Kothi” (men who tend to be receptive partners during sex), bisexual and transgender.

### Data management and statistical analysis

Data was collected using a structured questionnaire in hard copy. All data was entered into the database developed for the size estimation and analysis was done in Excel and SPSS 25.0. Initially, crude estimates of MSM were derived using the number of MSM active on the peak day and time of each of the five virtual sites as captured in Stage 2 for all 16 geo locations. Statistical adjustments were made to account for day and time variation as well as overlap among individuals with multiple profiles within a single virtual platform and multiple profiles on different platforms.

### Adjusting day and time variation and overlap between virtual sites

a. Adjusting day and time variation

Time-related adjustment was necessary as virtual sites users are not active all the time, every day. We used a correction factor determining the percentage of all MSM online on Sundays (peak day) and at night (peak time).


$$E=\frac{\sum_{i=1}^{n}\mathrm{Ei}}{p_{1}*p_{2}}$$

Where *E* is the adjusted estimate of all MSM active accounting for day and time; *Ei* is the unadjusted number of MSM active on virtual site i; *p*_*1*_ and *p*_*2*_ are the proportions of all interviewed MSM active as recorded on the peak day (Sunday) and peak time (night) respectively and *i = 1…n* is the number of virtual sites.

b. Adjusting overlap within and between virtual sites:

A second level of adjustment was required to account for individuals with multiple profiles within a single virtual site and multiple profiles on different sites. The unique number of active MSM was determined using the formula:

$$E_{f}=E*(1-p)+E*\frac{p}{m}$$

Where:

*E*_f_ is the adjusted estimate of individual MSM active on the virtual sites accounting for day, time and multiple profiles; *E* is the number of MSM active on all virtual sites adjusting for day and time variations; *p* is the proportion of MSM using more than one virtual site; *m* is the mean number of virtual sites a respondent uses, if he has more than one profile.

We used the 95% confidence interval of the mean, ‘*m*’, to estimate the lower and upper estimate of population size. Let *m* be the mean number of profiles, and *SE*_*m*_ be the standard error of the mean, then the lower and upper limit of mean number of profiles are derived as:

Lower limit (*m*_*l*_) = *m*—*Z*_a_*SE_*m*_; Upper limit (*m*_*u*_) = *m* + *Z*_a_*SE_*m*_

Where, Z_*a*_ = 1.96 is the Z value at 95% confidence level

The final adjusted lower and upper estimates of MSM on virtual sites is estimated as:

Lower estimate of MSM (*E*_*L*_), *E*_*L*_ = E * (1 –p) + E * (p/m_*u*_), Where *m*_u_ is the upper confidence limit of mean number of profiles.

Similarly, the upper estimate of MSM (*E*_*U*_), *E*_*U*_ = E * (1 − p) + E * (p/m_*l*_), where *m*_*l*_ is the lower confidence limit of mean number of profiles.

### Ethical considerations

The Sigma Institutional Review Board (10040/IRB/20-21), New Delhi granted ethics approval for the study. Informed consent discussions ensured that participants were aware that study involvement was voluntary and that non participation would have no impact regarding engagement with HIV treatment or prevention services. All data was de-identified and names of the virtual platforms were not made public.

## Results

### Size estimation and temporal patterns of platform use

**Fig 2:**
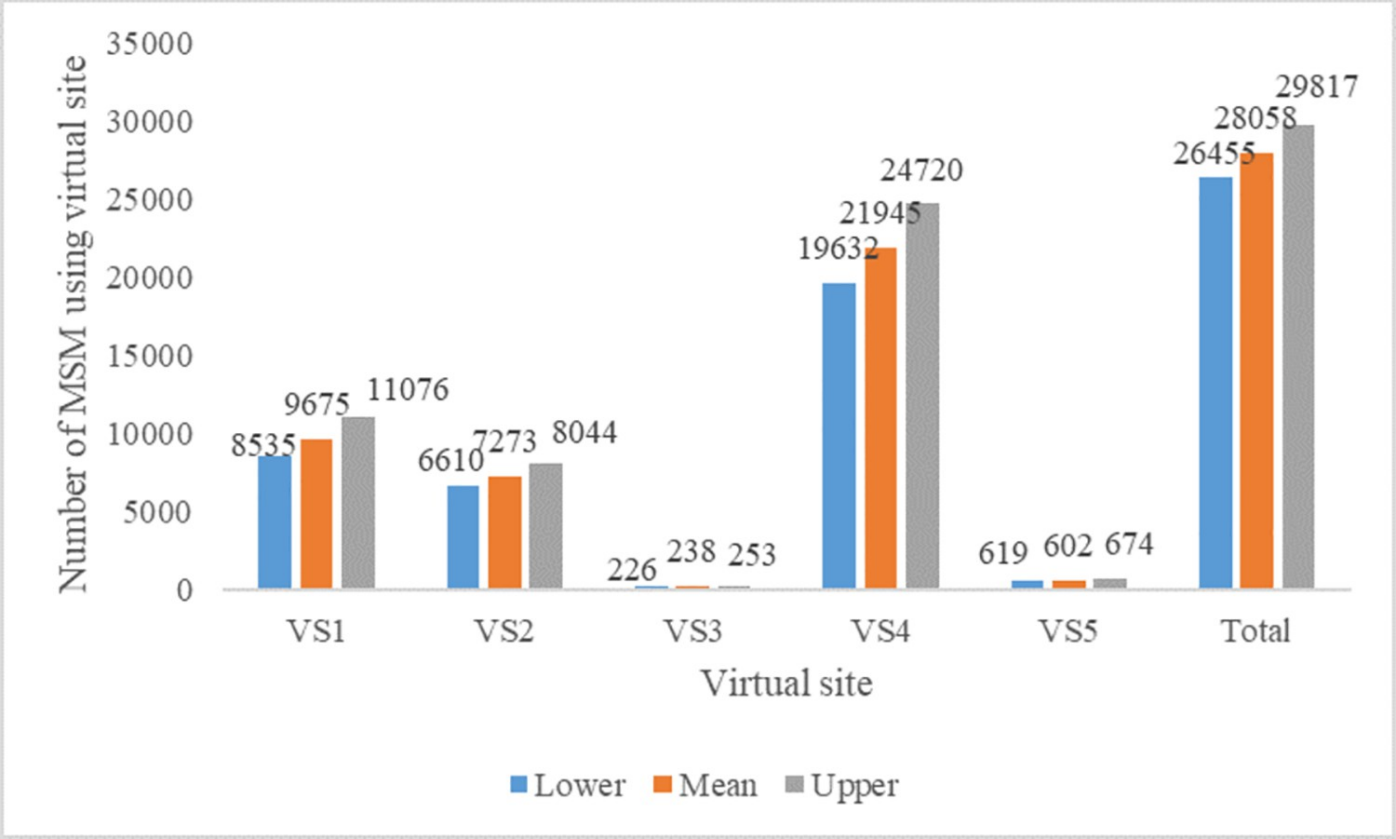
Number of MSM using different virtual sites.

**Fig 3:**
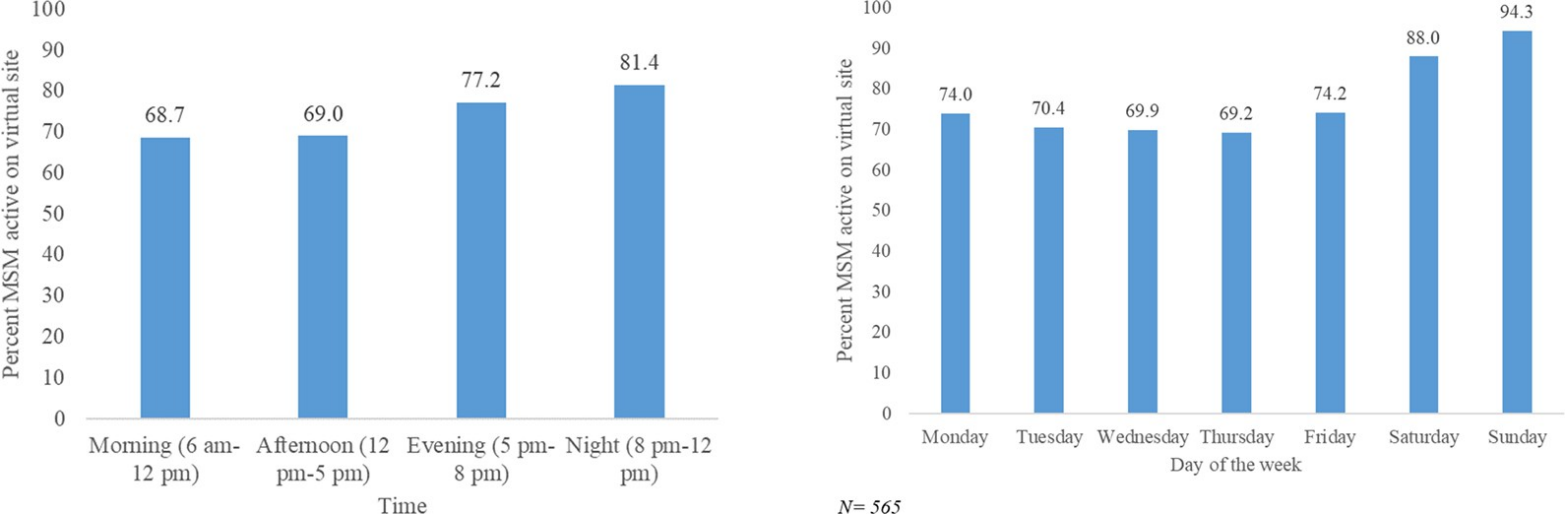
**a**: Temporal distribution of MSM using virtual sites by time of day. **b**: Temporal distribution of MSM using virtual sites by day of the week.

An estimated 28,058 MSM (95% CI: 26,455–29,817) use virtual sites to find sexual partners (Fig 2). Popularity differed between virtual sites (Fig 2). The most popular site had an estimated 21,945 MSM (95% CI: 19,632–24,720), accounting for 78.2% of the virtual MSM population in Delhi using virtual sites. The second most popular site had an estimated 9675 MSM (95% CI: 8,535–11,076) users, accounting for 34.5% of virtual MSM in Delhi on virtual sites. The least popular platform had an estimated 238 MSM (95% CI: 226–253), accounting for 0.8% (Fig 2). About half of MSM (50.8%) have more than one virtual profile with an average of 1.67 virtual profiles. The majority of users were active at night (81.0%) and on Sundays (94.0%) (Fig 3A and 3B). 42% of MSM using virtual sites in Delhi also visited a physical hotspot to meet sexual partners in the month prior to mapping.

**Fig 4:**
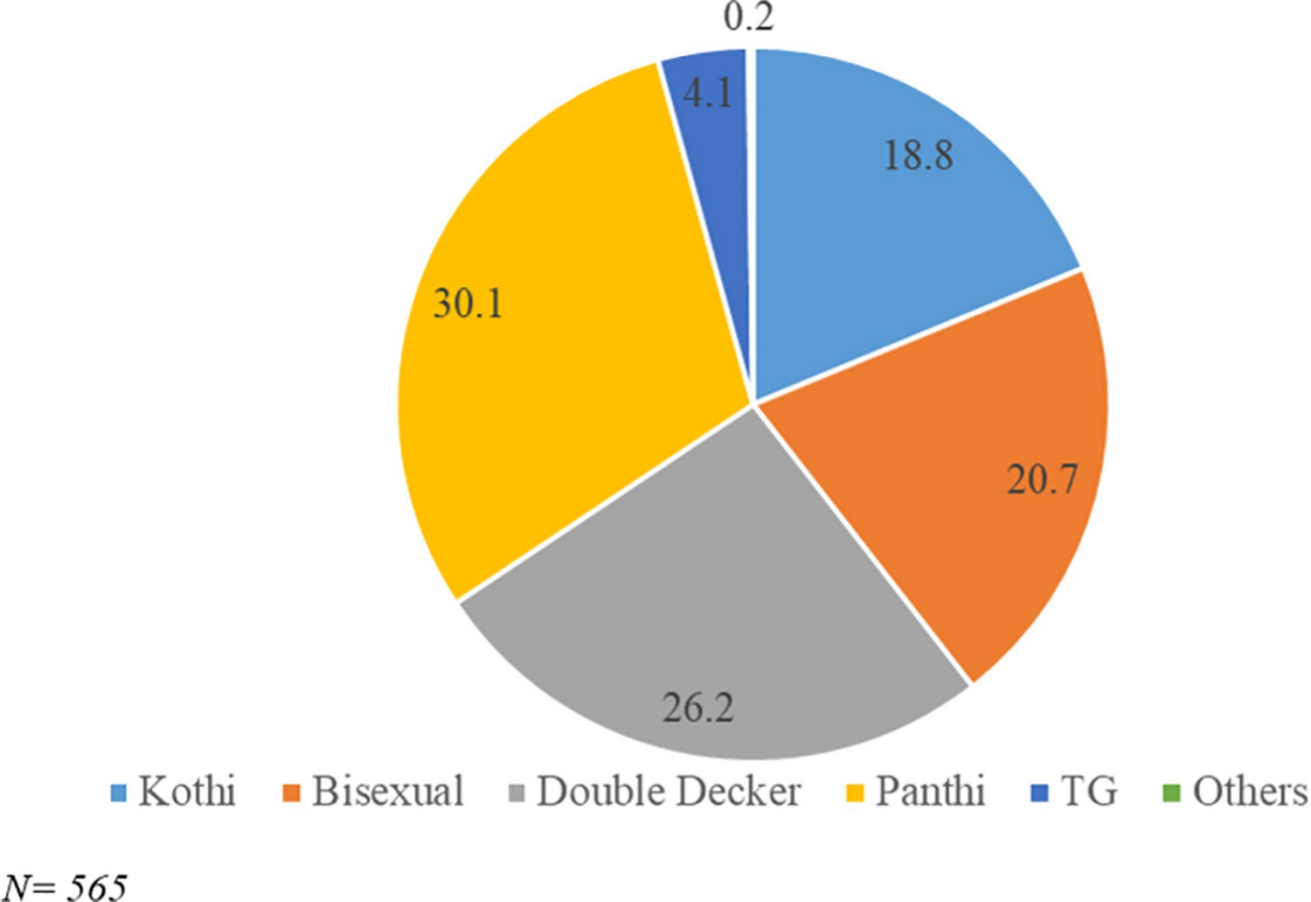
Subgroups of MSM active on virtual sites.

Of the 565 MSM participants active on virtual sites and interviewed, 30% identified themselves as “Panthi” (those who prefer an insertive role during sex), 26% self-identify as “double decker” (those who both insert and receive during sex), 21% self-identify as bisexual, 19% self-identify as “Kothi” (men who tend to be receptive partners during sex) and the remaining 4% self-identify as “Transgender or others” (Fig 4) [28].

### Program exposure of MSM active on virtual sites

**Fig 5:**
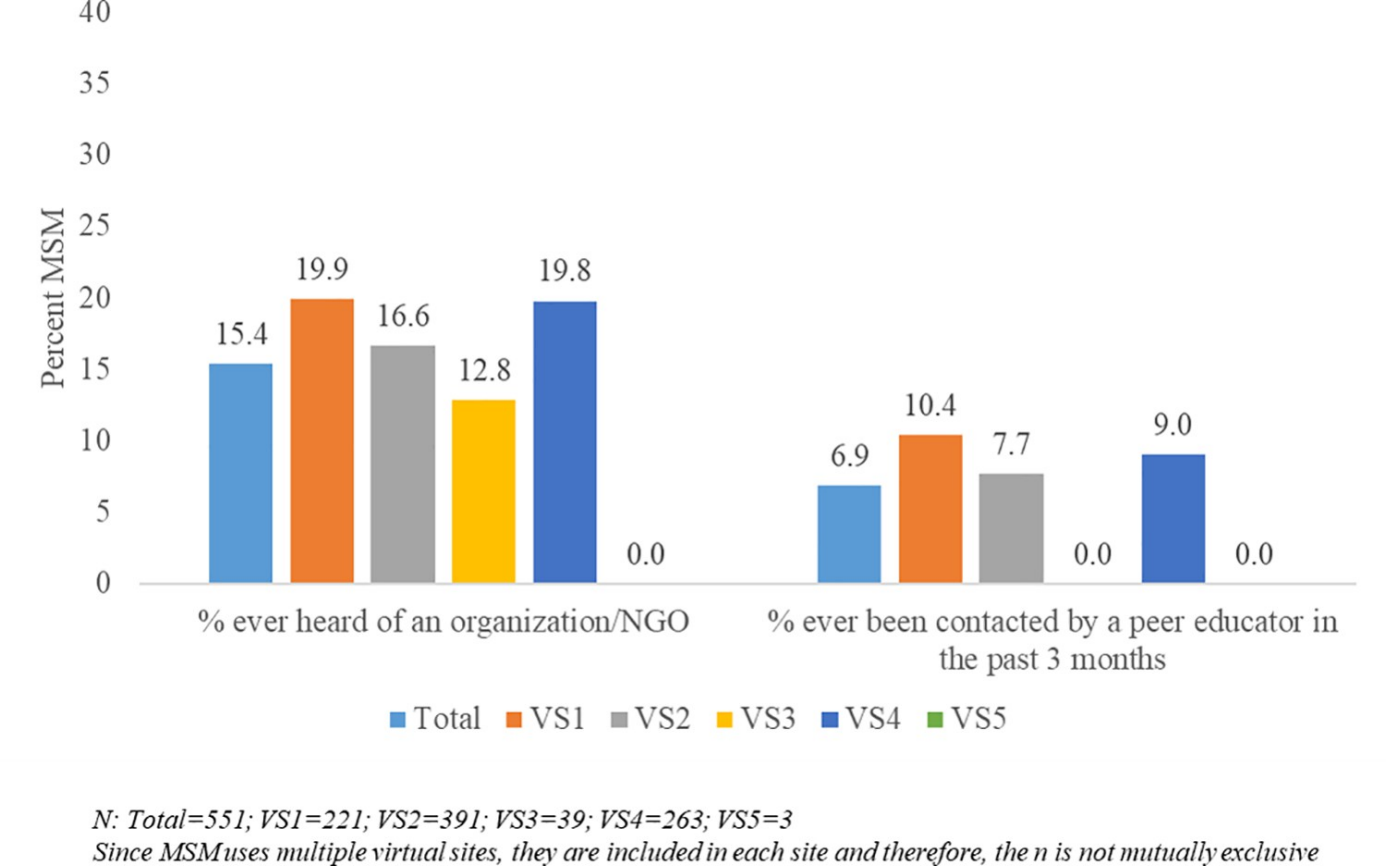
MSM on virtual sites who have heard of organizations providing HIV treatment and prevention services and those who have been contacted by a peer educator in the preceding 3 months by type of virtual site.

Only 15% of the total 565 respondents were aware of organisations providing HIV prevention and treatment services. Of these 565, only 7% had been contacted by peer educators in the three months before the mapping exercise (Fig 5). Among users of the most popular site, 20% of respondents were aware of organisations providing HIV prevention and treatment services and 9% had been contacted by peer educators in the preceding three months.

## Discussion

Size estimation of KP is essential for measuring program coverage and assessing the impact of public health interventions that address the HIV epidemic [29]. The conventional TI approach of intensive focus solely on physical hotspots excludes many MSM who exclusively seek sexual partners on virtual platforms. This paper demonstrates an approach to estimate size of MSM on virtual platforms. Further, the study demonstrates that only two-fifths of MSM who use virtual platforms visit physical hotspots. The failure of conventional TI programs in reaching MSM online was further illustrated by the small minority (7%) of respondents that had been contacted by peer educators in the three months preceding the mapping exercise and the low level of awareness of organizations providing HIV services. Since reaching all KPs is critical to program effectiveness, size estimations that do not include virtual platforms may lead to under-estimation of the MSM population and lower target-setting and programs designed to only reach MSM in physical hotspots will miss a significant number of MSM at high risk of HIV.

The patterns of virtual solicitation may be used to inform future areas of programmatic focus. For instance, usage patterns seem to vary with time and day, with greater activity during the night, and on Sundays. Further, a majority of the population was found to be active on just three virtual sites. These are important aspects related to the population, its availability and patterns in which people are connecting for sex, and can aid more effective planning of outreach activities. This information may also be used to identify peers within the MSM community who can act as champions and provide education about HIV services.

The virtual mapping methodology described here remains subject to several limitations. The size estimate is derived using the number of MSM active on virtual sites after accounting for overlap between and within virtual sites. Overlap is measured using the profiles of a random sample of MSM selected from the virtual sites, however this may underestimate the true extent of overlap. While the initial selection of respondents was random, the respondents who decided to participate in the quantitative interviews may be limited by selection bias. Furthermore, the data described here remains dependent on the quality of the data in the virtual platform: any inaccuracy within the virtual platform would transfer directly to our assessment. The data described above only applies to virtual platforms that met the feasibility criteria. Our data thus inherently excludes MSM operating on platforms that include heterosexual partnerships. Our data does not account for MSM who use social networking platforms and messenger groups that do not clearly demarcate the status of online users and an identifiable geo-location. Further virtual mapping studies that include social networking platforms and messenger groups may provide a more complete size estimate of this KP.

## Conclusion

Following the methodology described and considering the limitations mentioned, this virtual mapping exercise estimates that 28,058 MSM in Delhi (95% CI: range 26,455–29,817) use virtual platforms to seek sexual partners, of whom, 58% exclusively use virtual platforms. As such, this population is inadequately represented in the current TI programming and has a very low awareness of organizations that provide HIV treatment and prevention services. This signals a clear program gap that should be bridged. Virtual mapping must be incorporated into current public health programming to provide an accurate estimate of MSM at risk of HIV.
